# Identification of a TonB-Dependent Siderophore Receptor as a Novel Anti-Biofilm Target and Virtual Screening for Its Inhibitor in *Pseudomonas fluorescens* PF08

**DOI:** 10.3390/foods14030531

**Published:** 2025-02-06

**Authors:** Taizhi Shen, Changrong Cao, Ruiyu Zhu, Jian Chen, Feifei Wang, Yanbo Wang

**Affiliations:** 1 School of Biological and Chemical Engineering, Zhejiang University of Science and Technology, Hangzhou 310023, China; 2 Food Safety Key Laboratory of Zhejiang Province, School of Food Science and Biotechnology, Zhejiang Gongshang University, Hangzhou 310018, China; 3 School of Food and Health, Beijing Technology and Business University, Beijing 100048, China

**Keywords:** *Pseudomonas fluorescens*, TonB-dependent siderophore receptor, anti-biofilm, virtual screening, molecular dynamics simulation

## Abstract

*Pseudomonas fluorescens* is a vital food spoilage bacterium that commonly spoils foods in the biofilm state. Uncovering the targets responsible for biofilm formation and disrupting their function is a promising way to control bacterial biofilms and food spoilage. In this work, using the combination of qRT-PCR and construction of the gene deletion strain, Δ*tdsr*, TonB-dependent siderophore receptor D7M10_RS23410 was, for the first time, proven to play an essential part in the biofilm development of *P. fluorescens*. By utilizing structure-based virtual screening technology, a natural compound, adenosine monophosphate (AMP), with the highest binding activity to D7M10_RS23410, was obtained as an effective biofilm inhibitor. AMP significantly decreased the cell autoaggregation and biofilm biomass at sub-MIC concentrations (2.5, 1.25, and 0.625 mg/mL), mainly through inhibiting the generation of extracellular polymeric substances (EPS) in the biofilm matrix and promoting the cell motility. Furthermore, AMP was found to form hydrogen bonds with specific amino acid residues and stretched the protein structure of D7M10_RS23410, and this structural alteration undoubtedly interfered with the functionality of the D7M10_RS23410 protein.

## 1. Introduction

Microorganisms are determined to be the primary factor contributing to the deterioration of food during storage, transportation, and processing [1]. *Pseudomonas fluorescens* is a Gram-negative bacterium with a wide range of growth temperatures and is commonly found in ordinary and refrigerated foods, such as seafoods [2], meat [3], dairy products [4], and vegetables [5]. Moreover, *P. fluorescens* possesses proteolytic, lipolytic, and lecithinase activities; therefore, it is able to degrade foods to produce various substances, including trimethylamine, alcohols, esters, aldehydes, hydrocarbons, ketones, toluene, and sulfur-containing compounds [6], resulting in food spoilage.

Biofilms are assemblages of microbial cells that adhere to biotic or abiotic surfaces, embedding themselves within a matrix composed of extracellular polymeric substances (EPS), mainly consisting of proteins, polysaccharides (PS), and extracellular DNA (eDNA) [7], which endow bacteria with stronger resistance to external stress stimulation, serving as reservoirs for recurrent contamination, and thus offering an appropriate substrate for the growth of other bacteria, such as pathogens [8]. Notably, biofilms have significant involvement in the food industry. Microorganisms usually proliferate and form biofilms on various equipment, including pipelines, filters, and vessels, by utilizing excess nutrients from food residues that remain after processing. The formed biofilms will lead to blockages and fouling of the equipment and contaminate food products, ultimately bringing about food safety and quality issues [9]. The ability of *P. fluorescens* to form biofilms in food processing environments largely increases the chances of food spoilage, which has attracted widespread attention.

Iron is a necessary nutrient for the growth of bacteria and participates in numerous significant biological processes like electron transfer, cellular respiration, the citric acid cycle, and DNA synthesis [10]. However, iron mainly exists in an insoluble form in the natural environment, and it is difficult for bacteria to directly utilize it. TonB-dependent siderophore receptors are integral membrane proteins that play a crucial role in the uptake of iron in Gram-negative bacteria. These receptors facilitate the transport of siderophores, which are high-affinity iron chelators secreted by microorganisms to scavenge iron *Klebsiella pneumoniae* [11]. Recently, evidence has revealed that TonB-dependent siderophore receptors were engaged in the biofilm formation of *Klebsiella pneumoniae* [12] and *Pseudomonas fragi* [13]. Although there exist a large number of TonB-dependent siderophore receptor coding genes in the *P. fluorescens* genome, its role in *P. fluorescens* remain uncovered.

The escalating resistance of biofilms to traditional antibiotics or disinfectants has resulted in an increased interest in investigating novel strategies for combating biofilm formation [14]. Identifying effective anti-biofilm agents for *P. fluorescens* is an urgent priority for the food industry. However, the traditional means of finding biofilm inhibitors tend to be costly, time consuming, unpredictable, and lack efficiency; structure-based virtual screening (SB-VS) provides a more targeted and efficient approach, utilizing the three-dimensional configuration of the targets to screen a large database of compounds, potentially identifying those with high affinity and specificity for inhibiting biofilm formation. This approach can greatly minimize both the duration and expenses associated with the discovery process while increasing the likelihood of finding effective inhibitors [15]. Through this methodology, benzyl alcohol [16], (+)-catechin [17], guaiacol, and citral [18] were found to inhibit swimming ability and biofilm formation via their interaction with LuxI- and LuxR-type proteins. Based on the above information, it is supposed that the TonB-dependent siderophore receptor could be taken as the target to efficiently screen anti-biofilm compounds using the SB-VS method.

Herein, a TonB-dependent siderophore receptor, D7M10_RS23410, was determined to be significantly up-regulated in biofilm cells. Its vital role in controlling *P. fluorescens* PF08 biofilm formation was confirmed by the construction of a gene-knockout strain. Next, D7M10_RS23410 was taken as the target protein for the construction of a library of natural compounds containing 17,779 small molecules to obtain the biofilm inhibitor via SB-VS. Finally, the inhibitory influences of the screened molecule on biofilm-related phenotypes were evaluated, and the inhibiting mechanisms were investigated by utilizing molecular docking and dynamics simulation. This study helps provide not only a novel target and an anti-biofilm agent for biofilm control but also effective and practical screening methods for antibiofilm and antibacterial agents in the food industry.

## 2. Materials and Methods

### 2.1. Bacterial Strains

*Pseudomonas fluorescens* PF08, which was isolated from spoiled tuna in our previous study and stored in our lab, has been registered in a publicly available culture collection under the accession number CCTCC M 2020065. *P. fluorescens* PF08 was cultured in Luria–Bertani (LB) medium at 28 °C.

### 2.2. Biofilm Formation

An overnight culture of tested strains was diluted in LB broth at a ratio of 1:100 and then transferred into Petri dishes (no. 705001; Corning Incorporated, Corning, NY, USA) with 3 mL per sample and incubated for 24 h and 48 h at 28 °C. The free cells in the medium were collected. Biofilms were gently washed with phosphate-buffered saline (PBS, pH 7.2) and were then resuspended in 1.5 mL PBS using cell scrapers (Corning^®^ Costar^®^ cell scrapers 3010, Corning, NY, USA).

### 2.3. The qRT-PCR Analysis

RNA extraction was conducted following the methods outlined in prior studies [19]. RNA was extracted from 24-hour-cultured free cells and biofilm cells with the RNeasy mini kit (Qiagen, Hilden, Germany), respectively. For eliminating genomic DNA contamination, total RNA was treated with Turbo DNA-free (Thermo Fisher Scientific, Waltham, MA, USA). Afterwards, the concentration and purity of the RNA were tested using NanoDrop, and RNA integrity was assessed using agarose gel electrophoresis. cDNA synthesis was carried out with random hexamers (GE Healthcare, Uppsala, Sweden) alongside SuperScript II reverse transcriptase (Invitrogen, Carlsbad, CA, USA). The qRT-PCR was conducted following the previously established protocols [20]. Primers used are detailed in Table S1. The comparative levels of tested genes were standardized against the abundance of 16S rRNA.

### 2.4. Mutant Construction

Target genes’ in-frame deletion mutants were constructed as stated in a prior study [19,21]. The bacterial strains and plasmids utilized in this work are listed in Table 1, and the primers utilized are detailed in Table S1. Ultimately, colonies that were resistant to sucrose and sensitive to gentamycin were identified as deletion mutants through PCR screening, which was further validated via DNA sequencing.

### 2.5. Biofilm Quantification

The total biofilm was quantified through crystal violet staining, as previously described [21]. In brief, after incubation, biofilms were washed, air-dried, and dyed using 1% crystal violet (20 min). The stained biofilms were completely rinsed using sterile water and, once dry, were treated with 95% ethanol to dissolve the violet stain. The biofilm’s biomass was then measured with a spectrophotometer (SpectraMax^®^ i3x, Molecular Devices, Sunnyvale, CA, USA) at 595 nm.

### 2.6. Scanning Electron Microscopy

Biofilm structure was determined via scanning electron microscopy (SEM). Polystyrene slides (14 mm in diameter) were incubated within the 1:100 diluted overnight cultures then subjected to an incubation period of 24 h at a temperature of 28 °C. The slides were then rinsed with PBS and subsequently fixed in 2.5% glutaraldehyde at 4 °C overnight. These slides were washed three times with 0.1 M PBS for 15 min each time. Then, 1% osmium solution was utilized to fix these samples for 1 to 2 h. After washing with 0.1 M PBS three times, the samples underwent dehydration, using an ethanol solution with varying concentrations (30%, 50%, 70%, 80%, 90%, and 95%), for 15 min at each level. Following this, they were subjected to two interventions with 100% ethanol, each lasting for 20 min. Subsequently, the samples were submerged in a 1:1 mixture of ethanol and isoamyl acetate for 30 min before being exposed to topure isoamyl acetate for either 1 h or left to soak overnight. Samples underwent critical point drying for a duration of 5 h and were coated and observed under scanning electron microscopy.

### 2.7. Homology-Based Modeling and Model Assessment

The amino acid sequence of the D7M10_RS23410 protein was obtained through the NCBI database and was uploaded to SWISS-MODEL (https://swissmodel.expasy.org, accessed on 8 June 2023) for 3D protein structure building. The most homologous sequence (identity > 50%) of D7M10_RS23410 was selected as the template for homology modelling. Prior to docking, the removal of water molecules was carried out, followed by the addition of hydrogen atoms, and an energy minimization process was subsequently executed. The 3D structure was assessed by PROCHECK, Verfiy3D, and ERRAT in the SAVES server (http://services.mbi.ucla.edu/SAVES/, accessed on 8 June 2023).

### 2.8. Structure-Based Virtual Screening and Molecular Docking

Initially, the natural compound dataset was prepared by combining 39,991 molecules, relying on the TCM Database@Taiwan [23] and the MolPort Natural Products database. Compounds with molecular weights of less than 500 (17,779 molecules) were selected for docking. We employed blind docking in our virtual screening process [24]. Docking studies for the target protein, D7M10_RS23410, were performed via PyRx0.8 software [25]. The preparation of all ligands was accomplished through the Open Babel option incorporated within PyRx [26]. The natural compound with the highest binding activity was selected and purchased to verify its function, and the docking results were analyzed in Discovery Studio 2021 [27].

### 2.9. Minimum Inhibitory Concentration (MIC) Assay

The MIC of AMP against *P. fluorescens* PF08 was determined following the broth microdilution approach in accordance with prior methods [18]. The adenosine monophosphate (AMP) was purchased from Aladdin (Shanghai, China) and was supplemented into LB at an ultimate concentration of 10, 5, 2.5, 1.25, 0.625, and 0 mg/mL. A total of 300 µL of each medium was added to the Bioscreen microplate. The *P. fluorescens* PF08 overnight culture was adjusted to 10^6^ CFU/mL and 1:100 diluted into the plate well, OD600 of which was measured every 2 h using the automatic microbial growth curve analyzer Bioscreen C and incubated at a temperature of 28 °C for 24 h, shaking at a speed of 200 rpm. The MIC was identified as the lowest level of AMP at which no growth of bacteria was observed.

### 2.10. Effect of AMP on P. fluorescens PF08 Biofilm Formation and Morphology

An overnight culture of *P. fluorescens* PF08 was 1:100 diluted in LB broth supplemented with AMP at an ultimate concentration of 0.625, 1.25, and 2.5 mg/mL, respectively, then transferred into Petri dishes (no. 705001; Corning Incorporated, Corning, NY, USA) with 3 mL per sample and incubated at 28 °C for 24 h. Then, the effects of AMP on the biofilm biomass and morphology were determined according to methods described above.

### 2.11. Effect of AMP on Cell Autoaggregation

Autoaggregation was analyzed as previously described [28]. In brief, overnight cultures from LB broth supplemented with AMP (0.625, 1.25, and 2.5 mg/mL) were resuspended in PBS at pH 7.4 to achieve an OD600 at 1.0. Two milliliters from each sample were distributed into three separate tubes, and the initial OD600 was recorded. Then, the tubes were stored at room temperature without agitation. After standing for 24 h, 200 μL was gently taken from the top of the suspension for OD600 measurement. Each strain’s measurement was normalized to the initial OD600.

### 2.12. Effect of AMP on Biofilm EPS Production

The biofilms were prepared as detailed above. The biofilm matrix was separated via centrifugation at 10,000× *g* (Microfuge^®^ 20R Centrifuge, Beckman Coulter, Brea, CA, USA) after being resuspended in 1.5 mL PBS. The supernatants were passed through a MILLEX^®^ GP Filter Unit with a pore size of 0.22 μm (SLGP033RB, Merck Millipore, Darmstadt, Hesse, Germany) for further use.

The quantity of exopolysaccharide produced in the biofilm matrices was determined based on the phenol/sulfuric acid method, with glucose used as the reference, as previously described [29]. To measure the extracellular protein, biofilm supernatants were subjected directly to Bradford solution treatment (no. 23200; ThermoFisher, Waltham, MA, USA), and bovine serum albumin (BSA) was utilized to establish the standard curve. For the quantification of eDNA, a mixture was prepared by combining 100 μL supernatant with an equivalent volume of 2 μM SYTOX Green (catalog number S7020; ThermoFisher, Waltham, MA, USA) solution in PBS. Lambda DNA standard (no. SD0011; ThermoFisher, Waltham, MA, USA) was used for eDNA quantification. Fluorescence was measured using a spectrophotometer (SpectraMax^®^ i3x, Molecular Devices, Sunnyvale, CA, USA). The excitation wavelength was set at 465 nm, while the emission wavelength was set at 510 nm.

### 2.13. Effect of AMP on Cell Motility

A total of 2 μL of an overnight culture was inoculated into the semi-agar medium, incorporating 1.0% tryptone, 0.5% NaCl, and 0.3% agar [28]. The diameters of the swimming zone were recorded after 24 h culturing at 28 °C.

### 2.14. The Ligand–Protein Interaction Analysis

The inhibitory mechanism of AMP on the TonB-dependent siderophore receptor was evaluated by Discovery Studio 2021 and PyMOL2.4 [27]. The ligand–protein interactions were displayed in both 2D and 3D plots. To elucidate the inhibitory mechanism, AMP was docked with D7M10_RS23410, and the ligand–protein interaction was assessed.

### 2.15. Molecular Dynamics (MD) Simulation

MD simulation is an useful tool that is widely used for validating the conformational flexibility and structural stability of complexes [30]. Here, the simulation was conducted with the Gromacs (ver. 5.1.4) package [31]. The force field that we used for all the simulations is the standard CHAMM molecular mechanics [32].

To enable the expansion of the simulation box in every direction, periodic boundary conditions were applied. The protein was contained within a cubic enclosure, maintaining a minimum distance of 2 nm from the box’s edges, and it was solvated using the single point charge (SPC) water model. To achieve electrical neutrality in the simulation system, counterions (specifically Na+ and Cl−) were introduced. The minimization of energy was carried out through the steepest descent method, with a tolerance threshold set at 1000.0 kJ/mol/nm. Equilibration of the water and ions around the protein proceeded through two phases: first, a 100 ps run was conducted with constant volume and temperature (NVT); this was succeeded by a second phase of 100 ps, where the system was maintained at constant pressure and temperature (NPT). The system’s temperature and pressure stabilized at 297 K and 1 bar, respectively, utilizing the Parrinello–Rahman approach. A simulation time step of 2 fs was employed, with snapshots taken every 10 ps. The trajectory analysis was also conducted by GROMACS.

### 2.16. Statistical Assessments

All statistical analyses were performed in triplicate following a totally randomized experimental design. The variation among treatments was evaluated with a one-way analysis of variance (ANOVA) at a significance threshold set at *p* < 0.05, utilizing SPSS version 17.0. The findings are presented as means ± standard deviation (SD).

## 3. Results and Discussion

### 3.1. Identification of a TonB-Dependent Siderophore Receptor D7M10_RS23410 as P. fluorescens PF08 Anti-Biofilm Target

**Figure 1:**
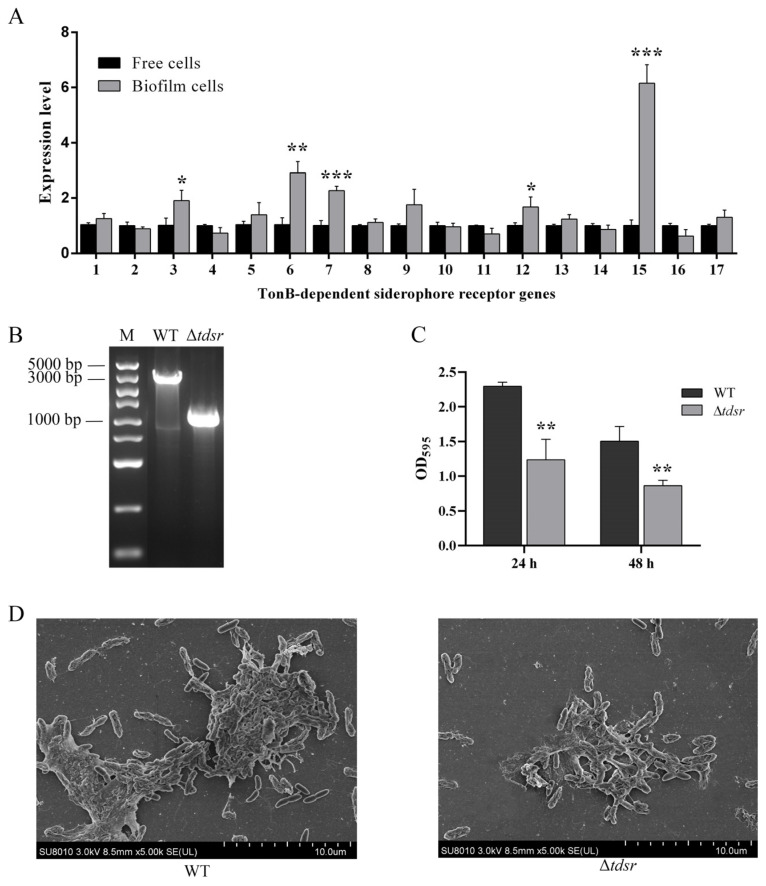
Identification of a TonB-dependent siderophore receptor D7M10_RS23410 as a *P. fluorescens* PF08 anti-biofilm target. (**A**) qRT-PCR of Tôn-dependent siderophore receptor coding genes of *P. fluorescens* PF08 free cells and biofilm cells. 1: *D7M10_RS00700*; 2: *D7M10_RS01265*; 3: *D7M10_RS01385*; 4: *D7M10_RS03530*; 5: *D7M10_RS04530*; 6: *D7M10_RS04775*; 7: *D7M10_RS10215*; 8: *D7M10_RS10260*; 9: *D7M10_RS10445*; 10: *D7M10_RS10450*; 11: *D7M10_RS10455*; 12: *D7M10_RS10800*; 13: *D7M10_RS15955*; 14: *D7M10_RS17460*; 15: *D7M10_RS23410*; 16: *D7M10_RS23460*; 17: *D7M10_RS25380*. (**B**) Agarose gel electrophoresis of *P. fluorescens* PF08 wild type (WT) and *D7M10_RS23410* in-frame deletion strain (Δ*tdsr*). (**C**) Biofilm biomass quantification using crystal violet method. (**D**) SEM images of biofilms formed by the WT strain and Δ*tdsr* strain; size bar: 10 μm. Data are displayed as the mean ± standard deviation (*n* = 3). * *p* < 0.05, ** *p* < 0.01, *** *p* < 0.001.

According to the genome information of *P. fluorescens*, 17 genes encoding for TonB-dependent siderophore receptor were found; that is, *D7M10_RS00700*, *D7M10_RS01265*, *D7M10_RS01385*, *D7M10_RS03530*, *D7M10_RS04530*, *D7M10_RS04775*, *D7M10_Rs0215*, *D7M10_RS10260*, *D7M10_RS10445*, *D7M10_RS10450*, *D7M10_RS10455*, *D7M10_RS10800*, *D7M10_RS15955*, *D7M10_RS17460*, *D7M10_RS23410*, *D7M10_RS23460*, and *D7M10_RS25380*. With the aim of figuring out their roles in *P. fluorescens* biofilm development, we first compared the transcription level of these genes in biofilm cells with that of free cells. Through qRT-PCR and statistical analyses, three TonB-dependent siderophore receptors, *D7M10_RS01385*, *D7M10_RS04775*, *D7M10_RS10215*, *D7M10_RS10800*, and *D7M10_RS23410*, were found to be significantly up-regulated in biofilm cells (Figure 1A). The results were similar to those found with respect to *Klebsiella pneumoniae* and *Pseudomonas fragi*, where the down-regulation of the TonB-dependent siderophore receptor was suggested to contribute to enhancing the diminishment of biofilm formation capacity [12,13].

In particular, the expression level of *D7M10_RS23410* was about 6-fold up-regulated in the biofilm cells compared to that of the free cells and was thus chosen as a representative for function verification. The *D7M10_RS23410* in-frame deletion mutant was successfully constructed and termed Δ*tdsr* (Figure 1B). As depicted in Figure 1C, biofilms formed by Δ*tdsr* at 24 h and 48 h was significantly reduced by 46.1% and 42.7%, respectively. In agreement with the decreased biofilms biomass, SEM images depicted that the biofilms formed by the Δ*tdsr* strain had a reduced number of attached bacterial cells and a diminished extracellular matrix content when contrasted with the wild type (Figure 1D). The findings indicate that the TonB-dependent siderophore receptor D7M10_RS23410 plays a vital role in *P. fluorescens* PF08 biofilm formation. These results were also supported by our previous study, in which it was found that siderophore plays a crucial part in the biofilm development of *P. fluorescens* [33], while TonB-dependent siderophore receptors were employed by bacteria to actively transport siderophore–iron chelates into the periplasm [34]. Reasonably, the TonB-dependent siderophore receptor D7M10_RS23410 can be considered as a promising target for biofilm controlling, which confirms our hypothesis.

### 3.2. Homologous Modeling and Model Assessment

**Figure 2:**
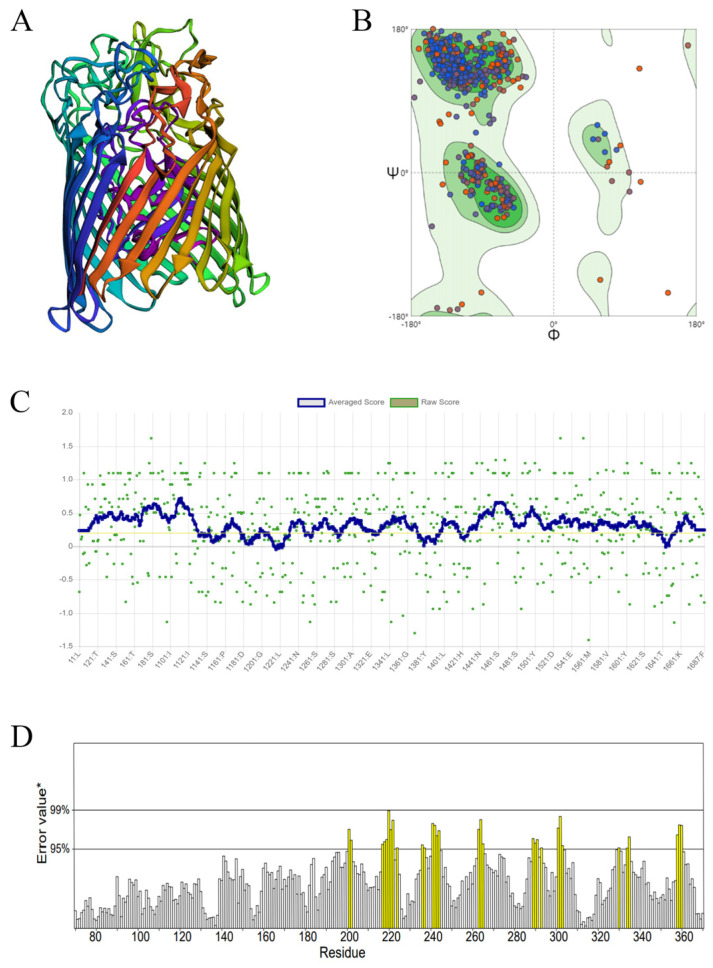
Homologous modeling and model assessment of D7M10_RS23410 protein: (**A**) The 3D structure of the D7M10_RS23410 protein; (**B**) the Ramachandran plot (blue dots represent amino acids in stable secondary structures, whereas red dots represent those in unstable secondary structures), (**C**) ERRAT, and (**D**) Verify −3D evaluation of D7M10_RS23410 3D structure (Amino acids with error values exceeding the 95% limit are statistically significant, marked yellow to indicate potential structural abnormalities).

It has been suggested that proteins with a sequence identity higher than 30% would have similar 3D structures [35]. The model–template alignment showed that the Ferric enterobactin receptor shared most identity (51.74%) with D7M10_RS23410, which guarantees the quality of the homology modeling. Therefore, the crystal structure of ferric enterobactin receptor was selected (complex with a competitive inhibitor, PDB: 3GAY) as the template, and the homology model of D7M10_RS23410 was constructed using the SWISSMODEL server (Figure 2A).

Moreover, the homology model quality was evaluated via PROCHECK, Verfiy3D, and ERRAT in the SAVES server. The analysis of the Ramachandran plot conducted by Procheck further validated the quality of the 3D model of D7M10_RS23410. The findings indicate that 90.4% of the residues were positioned within the most-preferred areas, while 8.1% fell into the additional allowed areas, 1.2% were located in the extensively allowed areas, and merely 0.3% resided in the disallowed areas (see Figure 2B). Specifically, most amino acid residues are in the stable states of the α-helix and β-sheet (Figure S1). The outcomes signify that the 3D structure of D7M10_RS23410 is suitable for future docking and virtual screening studies [35]. Additionally, the compatibility between the 3D atomic model and the 1D amino acid sequence was assessed, yielding an overall quality factor of 80.06% (>80% is considered acceptable) for Verify3D (Figure 2C) and 87.91% (>85%) is deemed acceptable for ERRAT (Figure 2D) in the SAVES server. The results from the Ramachandran plot, ERRAT, and Verify3D collectively indicate that the model we created is not only dependable but also maintains an excellent standard, making it appropriate for virtual screening research.

### 3.3. Virtual Screening

Virtually screening compounds against a specific receptor is an effective method for identifying the most promising candidates. In our research, the compounds were sourced from a natural compound database that includes 39,991 different molecules. Before proceeding with the docking of the small molecule into the three-dimensional structure of D7M10_RS23410 protein, we first prepared the system by establishing the grid and setting up the necessary parameters.

**Figure 3:**
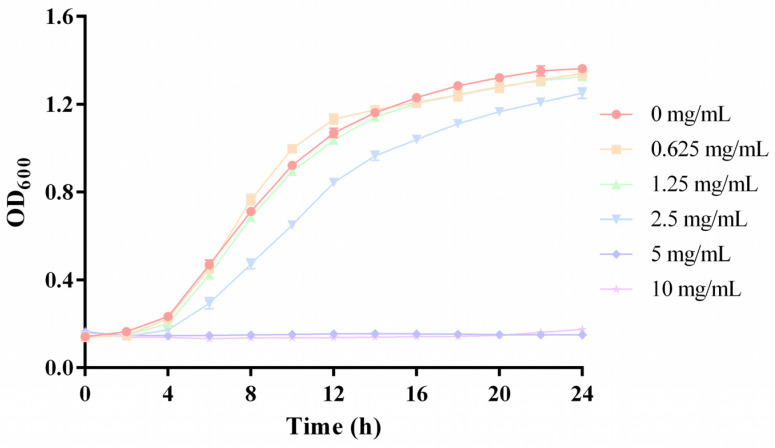
The growth curves of *P. fluorescens* PF08 dealt with different levels of AMP.

PyRx tools Autodock vina molecular docking program was utilized to conduct virtual screening; the outcomes were prioritized based on the binding energy, which illustrates the affinity of macromolecules and ligands [36]. These top eight compounds are listed in Table 2 according to their binding energy. The binding energy values of adenosine monophosphate (AMP), alantolactone, retinyl acetate, costunolide, cortodoxone, esculetin, deoxyarbutin, and schisandrin were −8.7 kcal/mol, −8 kcal/mol, −7.8 kcal/mol, −7.6 kcal/mol, −7.4 kcal/mol, −7.3 kcal/mol, −7 kcal/mol, and −7 kcal/mol, respectively. Among them, AMP had the highest binding activity and attracted our attention. AMP, referred to as a nucleotide, can be utilized as a food supplement aimed at enhancing immunological activity, particularly in infant formulas [37]. In addition, AMP can be used as a bitterness inhibitor, and it positively contributes to improving the acceptance and mouthfeel of food and beverages [38,39]. However, the anti-biofilm activity of AMP has not yet been revealed. The MIC of AMP against *P. fluorescens* PF08 was determined at 5 mg/mL (Figure 3), and the sub-MIC of AMP (2.5, 1.25, 0.625 mg/mL) was used for further investigation.

### 3.4. AMP Inhibited Biofilm Formation of P. fluorescens PF08

**Figure 4:**
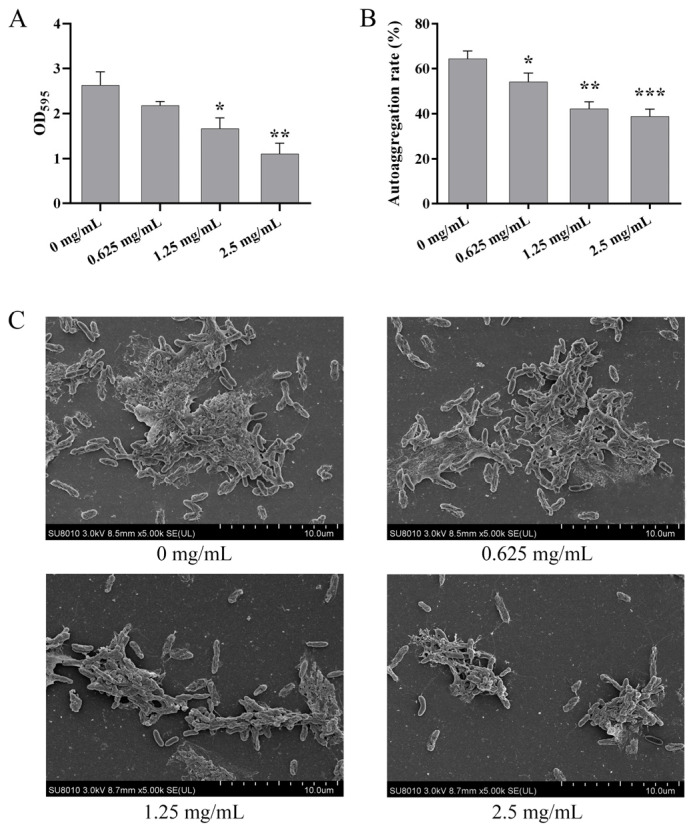
The effects of AMP on *P. fluorescens* PF08 biofilms: (**A**) inhibition effects on biofilm biomass quantification using crystal violet method; (**B**) inhibition effects on cell autoaggregation; (**C**) inhibition effects on the biofilm microstructure by SEM. Size bar: 10 μm. Data were displayed as the mean ± standard deviation (*n* = 3). * *p* < 0.05, ** *p* < 0.01, *** *p* < 0.001.

We investigated the influence of AMP on biofilm formation of *P. fluorescens* PF08 using crystal violet method. As presented in Figure 4A, AMP at Sub-MIC demonstrated concentration-dependent anti-biofilm activity against *P. fluorescens* PF08, and it decreased the biofilms by 28.86%, 36.72% (*p* < 0.05), and 57.94% (*p* < 0.01) when subjected to 0.625, 1.25, and 2.5 mg/mL AMP, respectively. This inhibitory effect of AMP on biofilm formation accorded with the effect caused by the deletion of D7M10_RS23410. This result also confirms our hypothesis that the TonB-dependent siderophore receptor could be taken as the target to efficiently screen anti-biofilm compounds using the SB-VS method.

### 3.5. AMP Inhibited Cell Autoaggregation

Bacteria and their surface elements can interact with each other to promote the aggregation among bacterial individuals, thereby facilitating the formation of biofilms [40]. After 24 h of standing, the autoaggregation of *P. fluorescens* PF08 dealt with AMP at dosages of 0.625, 1.25, and 2.5 mg/mL was suppressed by 15.76%, 34.44%, and 39.75%, respectively, when compared to the control (Figure 4B). The results imply that AMP can inhibit biofilm formation by inhibiting the cell autoaggregation of *P. fluorescens* PF08. The results from our research align with those of earlier studies, indicating that anti-biofilm could reduce cell autoaggregation [41,42].

### 3.6. AMP Impaired Biofilm Structure

The effect of AMP on *P. fluorescens* PF08 biofilm structure was determined via SEM. The structure of the biofilm, as verified via SEM imaging, is notably dispersed and lacks density, and it contains less extracellular matrix after AMP treatment, especially when treated with 1.25 and 2.5 mg/mL AMP (Figure 4C). These results are in accordance with the above biofilm biomass and cell aggregation results (Figure 4A,B), and they also align with earlier research suggesting that anti-biofilm agents have the potential to disturb the biofilm structure and weaken the adherence of microcolonies [41,43].

### 3.7. AMP Decreased EPS Production

**Figure 5:**
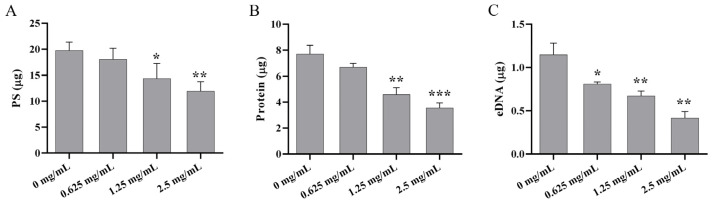
The effects of AMP on *P. fluorescens* PF08 biofilm EPSs production. The quantification of (**A**) PS, (**B**) protein, and (**C**) eDNA in the matrix of *P. fluorescens* PF08 biofilms after treatment with different concentrations of AMP. Data are displayed as the mean ± standard deviation (*n* = 3). * *p* < 0.05, ** *p* < 0.01, *** *p* < 0.001.

The biofilm matrix makes up more than 90% of biofilm dry mass, mainly including PS, protein, and eDNA, which can encapsulate the bacterial cells together and promote the maturation of biofilms [44]. As detailed in Figure 5, the contents of PS, protein, and eDNA in the biofilm matrix were all reduced with AMP treatment in a concentration-dependent manner. Specifically, PS decreased by 8.48%, 27.36%, and 39.71%; protein decreased by 15.23%, 40.38%, and 53.87%; and eDNA decreased by 29.51%, 41.43%, and 63.69% when treated with 0.625, 1.25, and 2.5 mg/mL AMP, respectively. These results imply that AMP can inhibit biofilm EPSs production and thus significantly interfere with biofilm formation. These results align with studies showing that anti-biofilm agents could reduce the levels of PS, proteins, and nucleic acids in the bacterial biofilms [41,45].

### 3.8. AMP Promoted Cell Motility

**Figure 6:**
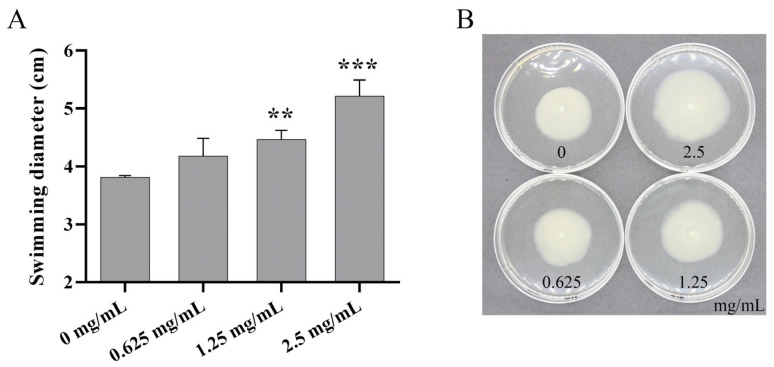
The effects of AMP on cell motility: (**A**) the swimming diameter; (**B**) the representative swimming zone images. Data are displayed as the mean ± standard deviation (*n* = 3). ** *p* < 0.01, *** *p* < 0.001.

Bacterial motility is crucial in the biofilm formation cycle, especially biofilm dispersion (Biofilm dispersion). As shown in Figure 6A, after 24 h of culturing, the swimming diameter of *P. fluorescens* PF08 treated with AMP at dosages of 0.625, 1.25, and 2.5 mg/mL increased by 9.62%, 17.24% (*p* < 0.01), and 36.92% (*p* < 0.001), respectively, indicating that AMP can promote cell motility and thereby inhibit biofilm formation. These findings align with earlier studies wherein anti-biofilm agents were found to facilitate biofilm dispersion by increasing bacterial motility, thus effectively preventing biofilm formation [46]. Moreover, our previous study revealed the negative relation between cell motility and biofilm formation in *P. fluorescens* PF08 [47].

### 3.9. Docking Analysis

**Figure 7:**
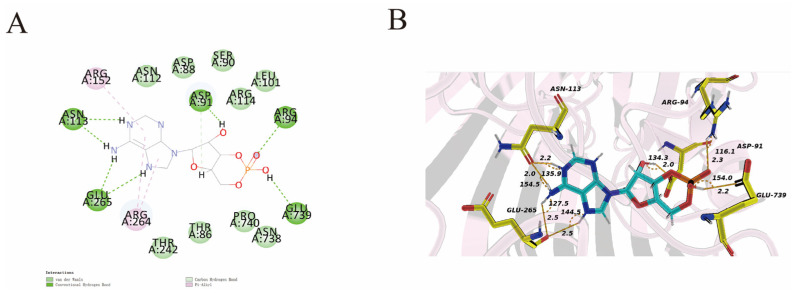
Two- and three-dimensional plots of ligand–protein interactions: (**A**) two-dimensional plots and (**B**) three-dimensional plots of the interaction between AMP and D7M10_RS23410 protein.

The protein–ligand interactions are illustrated in Figure 7. AMP docks into the active site of the D7M10_RS23410 protein. The interactions are illustrated by a 2D binding site diagram, which clearly describes and visualizes the characteristics of the ligand-binding site within the protein. Specifically, AMP predominantly forms typical hydrogen bonds with the D7M10_RS23410 protein, interacting with the residues of ASP91, ARG94, ASN113, GLU265, and GLU739. Specifically, if the bond distance is below 2.5 Å, it is considered strong; if it is within the range of 2.5–3.2 Å, it is categorized as moderate; additionally, a bond angle greater than 110° is deemed reasonable [48]. Consequently, AMP forms relatively strong hydrogen bonds with ASP91, ARG94, ASN113, and GLU739. Therefore, AMP forms relatively strong hydrogen bonds with the ASP91, ARG94, and ASN113 and GLU739.

### 3.10. Assessment of Molecular Dynamics Simulations

**Figure 8:**
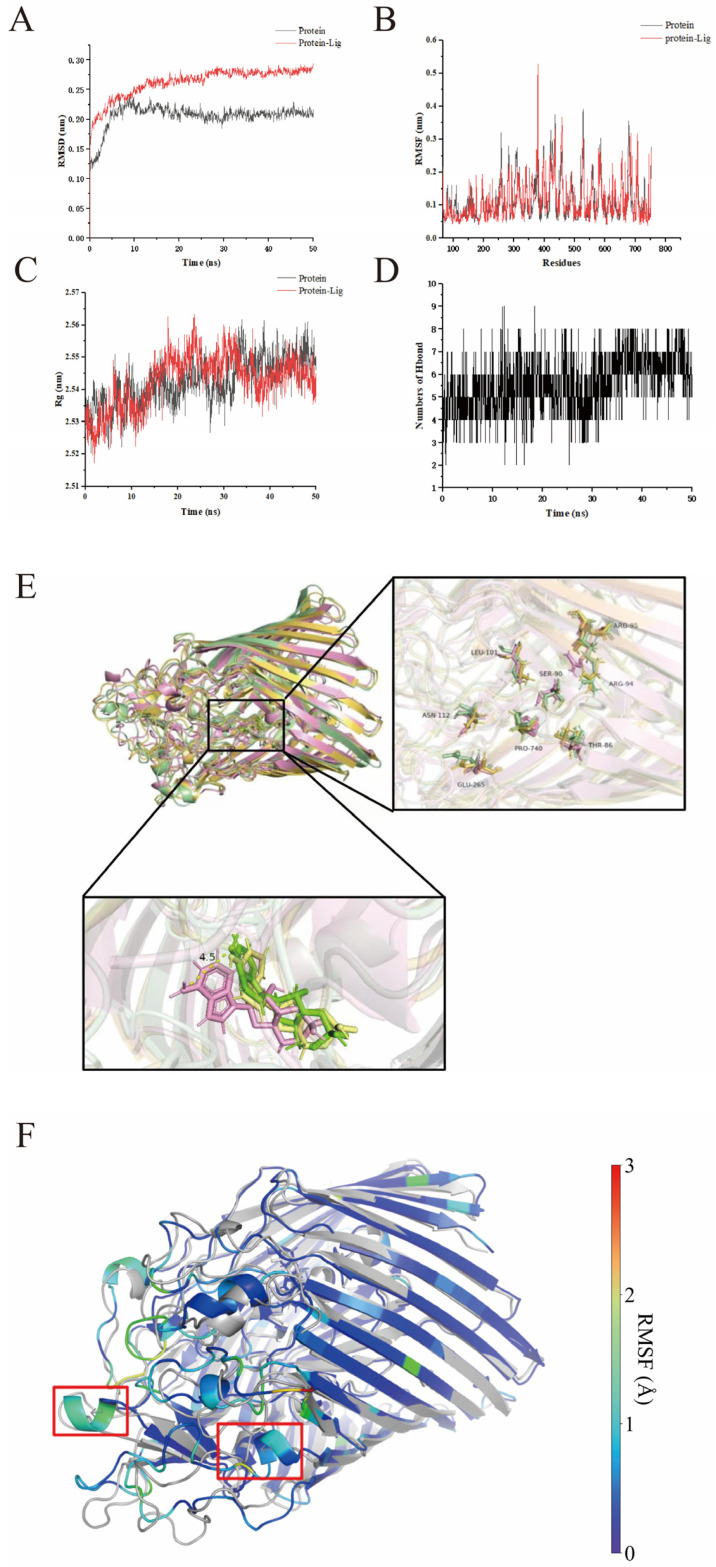
Molecular dynamics simulation analysis. The effects of AMP on the (**A**) RMSD value, (**B**) RMSF value, and (**C**) Rg value. (**D**) H-bonds number of the D7M10_RS23410 proteins during their interaction process. (**E**) Snapshot of the complex at 0 ns (pink), 20 ns (yellow), and 40 ns (green). (**F**) Superposition of representative conformations at 40 ns of MD simulation (colored according to RMSF values, with the initial conformation of the D7M10_RS23410 protein depicted in white).

As shown in Figure 8A, from 0 to 15 ns, the root mean square deviation (RMSD) values of the protein and complex increased, respectively, and then the fluctuation range of RMSD was within 0.1 nm, indicating that the binding complex achieved a stable phase, and that AMP could increase the stability of the D7M10_RS23410 protein.

Root mean square fluctuation (RMSF) is a key indicator when evaluating the flexibility of amino acid residues. During the binding process, several regions of the amino acid residues exhibited RMSF fluctuations (Figure 8B). Specifically, the RMSF values of the amino acid residues in regions such as 306–310, 375–381, and 678–679 fluctuated significantly. In particular, the fluctuations of residues 378, 379, and 677 were as high as 0.19 nm, 0.28 nm, and 0.19 nm, respectively. The data indicate that the binding of AMP to the D7M10_RS23410 protein causes the amino acid residues in these regions to behave more dynamically.

During the simulation, the compactness of the protein conformation is represented by the radius of gyration (Rg). After the binding of AMP to the D7M10_RS23410 protein, the contracted structure of the protein became more compact (Figure 8C), indicating that the binding of AMP to the D7M10_RS23410 protein is relatively stable.

Hydrogen bonds play a vital role in molecular recognition and assist in stable docked conformation between the compound and receptor [48]. In Figure 8D, with the extension of the reaction time, the number of hydrogen bonds formed between AMP and amino acid residues increased and gradually tended to be stable, which may be because AMP gradually found a new conformation and tended to be stable [49].

Structural superposition of the conformations at different time points (0 ns, 20 ns, and 40 ns) showed overall conformation of the protein, and the compound underwent relatively minor changes. Only some amino acid residues, such as LEU378 and ARG379, exhibited significant alterations, which is consistent with the RMSF results (Figure 8E). In Figure 8F, structural superposition of the representative conformation obtained from the MD simulations at 50 ns and the initial crystal structure revealed that two regions, highlighted in red, exhibited pronounced conformational changes, which indicates their important role in regulating the enzymatic activity of the D7M10_RS23410 protein [50].

The trajectory generated from the final 30 ns of the MD simulations was employed to calculate compound binding free energy using MM-PBSA. Among the four different binding energies, G_gas_, E*_vdW_*, and E_ele_ contribute much more than G_solv_, with G_gas_ being the most dominant term. The binding free energy of AMP is −12.6 kcal/mol (Table S2). In addition, LEU101 and ASN112 were the key residues, with the highest energy contributions of −1.1371 kcal/mol and −1.0211 kcal/mol, respectively (Figure S2). These results demonstrate that AMP can form relatively stable interactions with the active site residues of D7M10_RS23410.

According to the above result, it is suggested that structural modifications should be made to AMP to increase its binding free energy with the target protein and thus to enhance biological activity. For example, more functional groups capable of forming hydrogen bonds, such as an amino group (−NH_2_), can be rationally introduced into the AMP structure to increase the contribution of E_ele_.

## 4. Conclusions

Herein, a TonB-dependent siderophore receptor D7M10_RS23410 was first discovered to be responsible for the biofilm formation of *P. fluorescens* PF08. Reasonably, the TonB-dependent siderophore receptor was suggested to be an effective target candidate for biofilm controlling. Afterwards, AMP, known as a naturally occurring bitterness inhibitor, was selected as an inhibitor against D7M10_RS23410 using SB-VS technology and was verified to be useful in destroying biofilm formation by promoting cell motility, thus inhibiting biofilm EPSs production and cell autoaggregation, suggesting that AMP is a promising anti-biofilm agent for the treatment of biofilm-mediated food spoilage and contaminations. Moreover, SB-VS has proven to be a feasible approach for the identification of effective anti-biofilm compounds for *P. fluorescens* in this study, and it is expected to screen inhibitors for the growth and/or biofilm formation of other spoilage bacteria and pathogens in the food industry. However, this study has some limitations, including the need for further validation of the identified compound in real food matrices to assess its practical applicability in the food industry. Future research should focus on conducting application studies to evaluate the effectiveness of this compound in various food products.

## Figures and Tables

**Table 1:** Bacterial strains and plasmids used in this work.

Strains and Plasmids	Description	Reference or Source
*E. coli* strains		
*E. coli* DB3.1λ	Host for pHGM01	Originally from addgene
*E. coli* WM3064	Host for *pir*-dependent plasmids and donor strain for conjugation; RP4 (*tra*) in chromosome; Δ*dapA*	Originally from BioVector NTCC
*E. coli* BL21 (DE3)	Expression host	Originally from BioVector NTCC
*P. fluorescens* strains		
*P. fluorescens* PF08	Wild-type	Lab stock
Δ*tdsr*	*P. fluorescens* PF08 with *D7M10_RS23410* in-frame deletion	This study
Plasmid		
pHGM01	Apr, Gmr, Cmr; *mob*+; *att*-based suicide vector	[22]

**Table 2:** Screening results (top eight compounds) of D7M10_RS23410 protein inhibitor from the natural compound library.

Molecule Name	Molecular Formula	MW	Binding Energy (−kcal/mol)
Adenosine monophosphate	C_10_H_14_N_5_O_7_P	347.221	8.7
Alantolactone	C_15_H_20_O_2_	232.318	8
Retinyl acetate	C_22_H_32_O_2_	328.488	7.8
Costunolide	C_15_H_20_O_2_	232.318	7.6
Cortodoxone	C_21_H_30_O_4_	346.461	7.4
Esculetin	C_9_H_6_O_4_	178.141	7.3
Deoxyarbutin	C_11_H_14_O_3_	194.227	7

## Data Availability

The original contributions presented in the study are included in the article/Supplementary Materials. Further inquiries can be directed to the corresponding authors.

## Supplementary Materials

The following supporting information can be downloaded at https://www.mdpi.com/article/10.3390/foods14030531/s1: Table S1: Target genes and primers used in this study; Table S2: Binding free energies of the protein−ligand interactions; Figure S1: The topological structure of the protein; Figure S2: The contributions of key amino acid residues around AMP for the total binding free energies.
