# Supplementary material for: Identification of a TonB-Dependent Siderophore Receptor as a Novel Anti-Biofilm Target and Virtual Screening for Its Inhibitor in Pseudomonas fluorescens PF08
Source: Foods. 2025 Feb 6;14(3):531. doi: 10.3390/foods14030531 (PMC11816823; doi:10.3390/foods14030531)
Supplement: Supplementary file 1 [file foods-14-00531-s001.zip › foods-3400681-supplementary.pdf]

Table S1. Target genes and primers used in this study.

Purpose	Gene name	Primer / Sequence
**qRT-PCR**	D7M10_RS00700	Forward / CCTACACCGTCTACGATGCC
		Reverse / TGCACTGCGACACATAGGTT
	D7M10_RS01265	Forward / GTCAAAGACACCAACACCGC
		Reverse / GCGAATGCCATCGAGGTAGA
	D7M10_RS01385	Forward / TTTACAGCAGAGCTTCGGCA
		Reverse / GCTTTGCCCATACAGCATCG
	D7M10_RS03530	Forward / CCAAGGGCCAGTACAACCAT
		Reverse / GTTGTCGAAATAGGTCGCGC
	D7M10_RS04530	Forward / TTGAACGATGCCTGGAAGCT
		Reverse / TGCCGATATCGAACAGACCG
	D7M10_RS04775	Forward / TTTGAACGCGTTGCTCAAGG
		Reverse / GCGCTCGAATTCTTCACGAC
	D7M10_RS10215	Forward / AGGGCTACAACCTTTACGGC
		Reverse / GCTTGACCGTGTTGTTCCAC
	D7M10_RS10260	Forward / GGGTGCGCGATATCAACAAC
		Reverse / TCACCGTTGAGTTCCAGCTC
	D7M10_RS10445	Forward / AGCGTGCTCTACAACGTCAA
		Reverse / CGTAGGGTCAGCATGTGGTT
	D7M10_RS10450	Forward / ATGGCTCCGGCATTGGTTTA
		Reverse / GATCCCTGTTCGCTGCGATA
	D7M10_RS10455	Forward / GGCGCGTAGTAGTAGCCAAT
		Reverse / ACTTGGCGAAGACTTCCTGG
	D7M10_RS10800	Forward / TGGGATTTGGTCGACAGTGG
		Reverse / GAGAGTTTATCCGCGCGGTA
	D7M10_RS15955	Forward / GGAGTACGGCACCTTCAACA
		Reverse / GTCGGCATCATCGAACAAGC
	D7M10_RS17460	Forward / CTTCGGCACTAGCGCATTTC
		Reverse / CACGGTTTGCCATTGTTCGT
	D7M10_RS23410	Forward / CTACAAGACGCCCAACCTGT
		Reverse / GGTGTTGCTGGCGTTGATTT
	D7M10_RS23460	Forward / TCCACCGGCCTGGAATTTAC
		Reverse / GATCGCTCAATGACGTGCAC
	D7M10_RS25380	Forward / GACGCGGTCAAATACATGCC
		Reverse / GTAGGGCGAGACGATCAAGG
**Mutant construction**	D7M10_RS23410	5’O / GGGGACAAGTTTGTACAAAAAAG CAGGCTGTCGTATTCATCCGGGTCGC
		5’I / GGTCCGGGTTCGCTATCTATGTGGC GCGACATGAAATTCC
		3’I / ATAGATAGCGAACCCGGACCCCACT TCTAAGGCGCTAGACG
		3’O / GGGGACCACTTTGTACAAGAAAGC TGGGTGGTGCAGTTGCAGCATCAG

Table S2. Binding free energies of the Protein−Ligand interactions.

ligand	E_{vdW} (kcal/mol)	E_{ele} (kcal/mol)	ΔG_{gas} (kcal/mol)	ΔG_{solv} (kcal/mol)	ΔG_{bind} (kcal/mol)
AMP	-30.6±3.5	-87.6±8.9	-118.2±8.5	105.5±8.9	-12.6±6.5

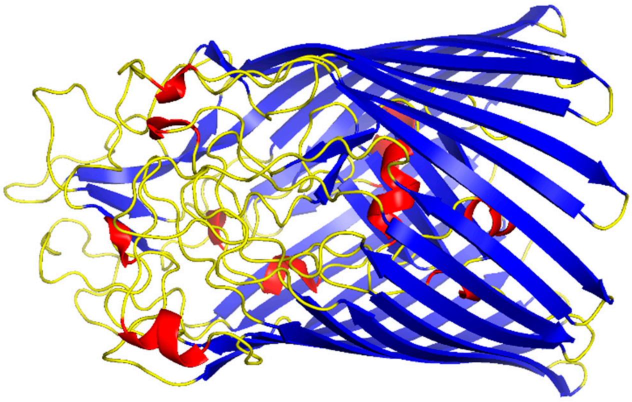

Figure S1. The topological structure of the protein. Blue represents α-helix, red represents β-sheet, and yellow represents random coil and other structures.

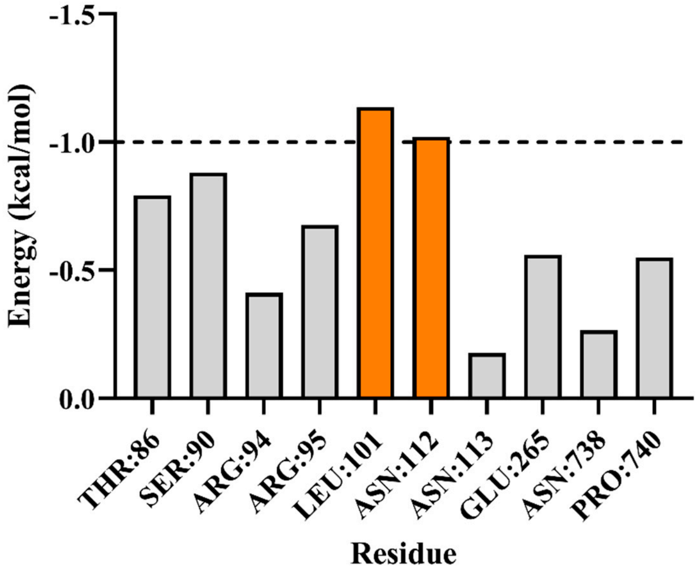

Figuref S2. The contributions of key amino acid residues around AMP for the total binding free energies in the interaction of AMP with D7M10_RS23410
